# Cadherins mediate sequential roles through a hierarchy of mechanisms in the developing mammillary body

**DOI:** 10.3389/fnana.2015.00029

**Published:** 2015-03-19

**Authors:** Nora-Emöke Szabó, Roberta Haddad-Tóvolli, Xunlei Zhou, Gonzalo Alvarez-Bolado

**Affiliations:** ^1^Department Neurobiology and Development, Neural Circuit Development Unit, IRCMMontréal, QC, Canada; ^2^Department of Neuroanatomy, University of HeidelbergHeidelberg, Germany

**Keywords:** neuronal birthdates, cell sorting, combinatorial, differential adhesion, mamillary body

## Abstract

Expression of intricate combinations of cadherins (a family of adhesive membrane proteins) is common in the developing central nervous system. On this basis, a combinatorial cadherin code has long been proposed to underlie neuronal sorting and to be ultimately responsible for the layers, columns and nuclei of the brain. However, experimental proof of this particular function of cadherins has proven difficult to obtain and the question is still not clear. Alternatively, non-specific, non-combinatorial, purely quantitative adhesive differentials have been proposed to explain neuronal sorting in the brain. Do cadherin combinations underlie brain cytoarchitecture? We approached this question using as model a well-defined forebrain nucleus, the mammillary body (MBO), which shows strong, homogeneous expression of one single cadherin (*Cdh11*) and patterned, combinatorial expression of *Cdh6*, *−8* and *−10*. We found that, besides the known combinatorial *Cdh* pattern, MBO cells are organized into a second, non-overlapping pattern grouping neurons with the same date of neurogenesis. We report that, in the *Foxb1* mouse mutant, *Cdh11* expression fails to be maintained during MBO development. This disrupted the combination-based as well as the birthdate-based sorting in the mutant MBO. *In utero* RNA interference (RNAi) experiments knocking down *Cdh11* in MBO-fated migrating neurons at one specific age showed that *Cdh11* expression is required for chronological entrance in the MBO. Our results suggest that neuronal sorting in the developing MBO is caused by adhesion-based, non-combinatorial mechanisms that keep neurons sorted according to birthdate information (possibly matching them to target neurons chronologically sorted in the same manner). Non-specific adhesion mechanisms would also prevent cadherin combinations from altering the birthdate-based sorting. Cadherin combinations would presumably act later to support specific synaptogenesis through specific axonal fasciculation and final target recognition.

## Introduction

The mammalian brain is formed by a large variety of neuronal aggregates organized as layers, nuclei and subnuclei. The diversity of forms found in animal tissues is considered to be largely the result of conserved morphogenetic processes and mechanisms (Lecuit, 2008). If and how these underlie brain histogenesis is not well understood. Differential cell-cell adhesive interactions are essential drivers of morphogenesis (Edelman, 1988). Classical cadherins are transmembrane proteins mediating cell-cell adhesion with roles in cell sorting and in axonal connectivity (Takeichi, 2007). The intriguing combinatorial cadherin expression patterns in brain regions (see for instance (Hertel et al., 2008, 2012; Krishna-K et al., 2011)) have been proposed (Redies and Takeichi, 1996) to underlie the sorting of specific neuronal subpopulations. As an additional function, a combinatorial mechanism underlying appropriate connectivity/synaptogenesis has been suggested (Suzuki et al., 1997; Bekirov et al., 2002; Treubert-Zimmermann et al., 2002) since, in some systems, projecting neurons express the same cadherin combinations as their targets.

If combinations of cadherins confer adhesion specificity (or synaptic specificity), homophilic adhesion (e.g., Cdh11 would bind only, specifically, to Cdh11) would be indispensable. Only in that way could combinations specifically recognize each other. Data from a variety of experimental systems has proven the importance of homophilic binding of one cadherin (not a combination) in morphogenesis (Gumbiner, 2005; Suzuki and Takeichi, 2008), axonal fasciculation (Treubert-Zimmermann et al., 2002), synapse formation (Manabe et al., 2000; Elia et al., 2006; Paradis et al., 2007; Suzuki et al., 2007) and guidance of migrating neurons (Luo et al., 2004). The role of cadherin combinations in neuronal sorting has been experimentally proven in chicken hindbrain motoneurons (Astick et al., 2014). Still, that cadherin combinations form a specific code underlying brain histogenesis is far from clear.

To complicate things, the study of cell sorting phenomena in tissue aggregates *in vitro* suggests an additional, non-molecularly-specific source of histogenetic order. This consists of physical forces like the surface tension of cell aggregates, resulting from the ratio between adhesion and cortical tension (Steinberg, 1962a,b,c; Krieg et al., 2008; Manning et al., 2010). Indeed, non-specific adhesion differentials can mediate cadherin-dependent cell sorting in culture (Steinberg and Takeichi, 1994; Duguay et al., 2003) and determine the antero-posterior body axis of the Drosophila embryo (Godt and Tepass, 1998; González-Reyes and St Johnston, 1998). This paradigm presupposes heterophilic binding and, consistently, cadherins exhibit actually little binding specificity (Shimoyama et al., 1999, 2000; Niessen and Gumbiner, 2002; Foty and Steinberg, 2005; Prakasam et al., 2006; Krieg et al., 2008; Shi et al., 2008). However, if this paradigm can be applied at all to migrating neurons in the developing brain is open to question, and the possible role of non-specific adhesion forces in brain histogenesis has to our knowledge never been approached.

In summary, the questions of the actual role of the intricate cadherin combinations in brain cell sorting, and the relative importance of specific (homophilic) vs. non-specific (heterophilic) mechanisms are still mysterious.

**Figure 1:**
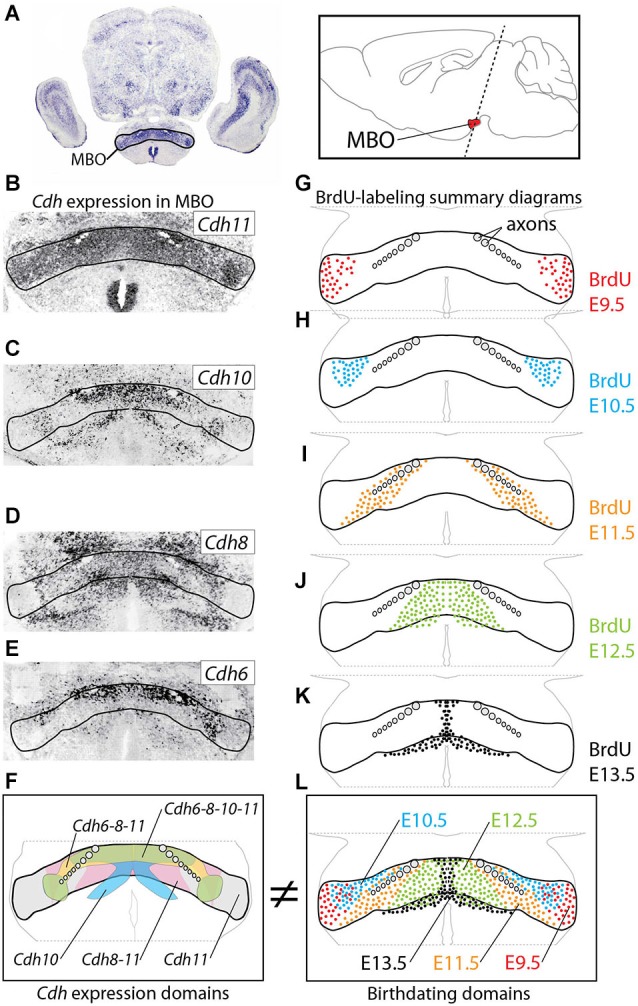
**Age of neurogenesis and Cadherin expression do not match. (A)** The MBO in a transverse section of E18.5 mouse brain labeled with *in situ* hybridization for Cdh11. Inset: position of the MBO on a sagittal diagram of the mouse brain, rostral to the left. **(B–E)** *In situ* hybridization (ISH) for cadherins (as indicated) on transverse sections of E18.5 MBO. **(F)** Summary diagram of cadherin expression in the MBO after the data in **(B–E)**. **(G–K)** Diagrams of transverse sections through E18.5 MBO labeled with anti-BrdU antibody after BrdU injection at E9.5 **(G)**, E10.5 **(H)**, E11.5 **(I)**, E12.5 **(J)** and E13.5 **(K)**. **(L)** Summary diagrams of BrdU-labeled cells corresponding to the data in **(G–K)**.

Here we have tested in the developing mouse brain *in utero* the role of cadherins on neuronal aggregation. Our model is the developing mammillary body (MBO), a large, compact and well-delimited paired neuronal structure with defined functions (Vann and Aggleton, 2004) located in the hypothalamus (Figure 1A) and showing ubiquitous expression of* Cdh11* and patterned expression of *Cdh6*, *8*, and *10* (Kimura et al., 1996; Suzuki et al., 1997). Each MBO is medio-laterally subdivided into medial and lateral mammillary nuclei (Allen and Hopkins, 1988). We first explored the relation between neuronal birthdate and specific cadherin expression in MBO neurons. Then we analyzed cell sorting upon loss of *Cdh11* expression over the entire MBO during development. Finally, we used *in utero* electroporation and RNAi to reduce *Cdh11* expression in all MBO neurons born at a certain specific age and analyzed their position several days later.

Our results suggest that neuronal sorting inside brain nuclei is caused by adhesion-based, non-combinatorial mechanisms that keep neurons sorted according to birthdate information matching them to target neurons chronologically sorted in the same manner. Non-specific adhesion mechanisms would also prevent cadherin combinations from altering the birthdate-based sorting. Cadherin combinations would presumably act later to support specific synaptogenesis through specific axonal fasciculation and final target recognition.

## Materials and Methods

### Mice

Animals were housed and handled in ways that minimize pain and discomfort, in accordance with German animal welfare regulations (TierSchG) and in agreement with the European Communities Council Directive (2010/63/EU). The authorization for the experiments, including *in utero* electroporation, was granted by the Regierungspräsidium Karlsruhe (state authorities) and the experiments were performed under surveillance of the Animal Welfare Officer responsible for the Institute of Anatomy and Cell Biology. To obtain embryos, timed-pregnant females were sacrificed by cervical dislocation; the embryos were decapitated.

Wild type observations and electroporation experiments were carried out on C57BL/6 mice. Additionally, two mouse lines carrying null mutations of *Foxb1* were used, the *Foxb1-tauLacZ* (Alvarez-Bolado et al., 2000a), with beta-galactosidase as reporter, and the *Foxb1-Cre-GFP* (Zhao et al., 2007), with green fluorescent protein (GFP) as reporter. By crossing heterozygotes of both lines, Foxb1 homozygous mice were generated carrying one b-galactosidase-expressing Foxb1 null allele and one GFP-expressing Foxb1 null allele. In this way, the homozygotes as well as half of the heterozygotes carried only one b-galactosidase-expressing allele and so the intensity of beta-galactosidase expression could be compared between them in order to evaluate the size and shape of the MBO (see below).

### Size and Shape Measurements of the MBO

The brains of E18.5 homozygotes and beta-galactosidase-expressing heterozygote embryos (see above) were collected (three brains per age and genotype), embedded in agarose and cut sagittally with a vibration microtome into 100 μm thick sections. The sections were stained with the X-gal reaction (Zhao et al., 2007), then fixed and photographed. The sections were assigned to one of four medio-lateral regions of the MBO, and the section area (in arbitrary units) labeled by the X-gal reaction in the mammillary region was measured with Cell-F software (Olympus Soft Imaging Solutions, Münster, Germany). The combined section areas for every medio-lateral region were used as a proxy for the size of the region.

### Cell Density Measurement in the MBO

Twenty five μm thick sections of E18.5 Foxb1-Cre-GFP homozygous and heterozygous brains were labeled with the nuclear marker 4’,6-diamidino-2-phenylindole (DAPI) as well as an anti-GFP antibody to specifically stain the MBO. Two square regions (100 μm side) were defined in the medial and in lateral part of the MBO and the number of cells in each of them was counted by the optical dissector method (Coggeshall and Lekan, 1996).

### *In Utero* Electroporation

We have described the procedure in detail elsewhere (Haddad-Tóvolli et al., 2013). Timed-pregnant (E12.5) mice were anesthetized and the uterus surgically exposed. Plasmid encoding small hairpin RNAs (shRNA) (1,5 μg/μl) (see below) was mixed with pCAGGS-GFP reporter vector (0,8 μg/μl), and approximately 1 μl of this DNA mixture was injected with a pulled micropipette into the third ventricle of each embryonic brain. Five pulses of square-wave current were applied (50 V, 50 ms on, 950 ms off) to each injected embryonic brain using a CUY21EDIT electroporator (Nepagene), and the pregnant mice were allowed to recover. The embryo brains were collected at E18.5 and those showing strong fluorescence in the mammillary region were prepared for further analysis. Some brains were fixed in 4% paraformaldehyde for 1–2 h at RT, embedded in gelatine-albumin and cut into 100–200 μm thick sections. The sections were then analyzed under a fluorescent microscope. Some brains were cryostat-sectioned at 20 μm for immunohistochemistry.

### Immunohistochemistry

We followed a published protocol (Szabó et al., 2011) on paraffin sections (15 μm). We used the following antibodies: anti-Cadherin11 (1:80) (monoclonal, Zytomed), anti-GFP (1:1000) (rabbit polyclonal, Invitrogen) and (1:500) (rabbit polyclonal, Abcam), anti-beta Galactosidase (1:500) (polyclonal, Abcam), anti-nestin (1:200) (monoclonal, Chemicon), anti-2H3 (1:5) (Developmental studies Hybridoma bank, monoclonal). Then we photographed the results with a Leica TCS SP5 confocal microscope.

### RNA Interference Plasmids

DNA plasmids encoding shRNA designed to interfere with Cdh11 mRNA were purchased from Sigma (NM_009866). The following three were tried in culture:

- shRNA-2 (1628s1c1) CCG GCC AAG TTA TAT CCA TGA AGT TCT CGA GAA CTT CAT GGA TAT AAC TTG GTT TTT G;
- shRNA-3 (1853s1c1) CCG GGC AGA AAT TCA CAA CAG ACA TCT CGA GAT GTC TGT TGT GAA TTT CTG CTT TTT G;
- shRNA-4 (2045s1c1) CCG GCC AAG ATT TAT CTT CAG CCT ACT CGA GTA GGC TGA AGA TAA ATC TTG GTT TTT G.

Successful interference (see below) was obtained with shRNA-3.

### Quantitative PCR Control of RNAi in Culture

HEK293T cells were plated (200,000 cells per 3.5 cm well). After 24 h in culture they reached 50% confluence and were transfected with one of the shRNA plasmids (either shRNA-2, -3 or -4, see above) plus a “target and control” plasmid carrying CAG promoter—*mCdh11* cDNA—IRES—EGFP—poly A—SV40 promoter—*neomycine phosphotransferase II* (*neo*)—poly A. A total of 2 μg of DNA per well were transfected (1.8 μg of shRNA plasmid plus 0.2 μg of “target and control” plasmid). Forty eight hours after transfection RNA was extracted, treated with DNAse I and reverse transcribed with the Superscript kit (Invitrogen) (2 μg RNA per reaction). The RNA was quantitated by PCR (StepOne Plus, Applied Biosystems) using the *neo* transcript to normalize. The transfections were done in triplicate and the quantitative RT-PCR was repeated three times per transfection.

### RNAi Complementation (“Rescue”) Experiments

A complementation construct was cloned carrying a human *CDH11* cDNA and the GFP reporter under the control of the CAG promoter (Niwa et al., 1991; Figures 11A,B). We performed this deletion on human CDH11 cDNA, whose nucleotide sequence is not 100% identical with the mouse *Cdh11*, to maximize the probability of the complementation construct not to be recognized by the shRNA.

**Figure 2:**
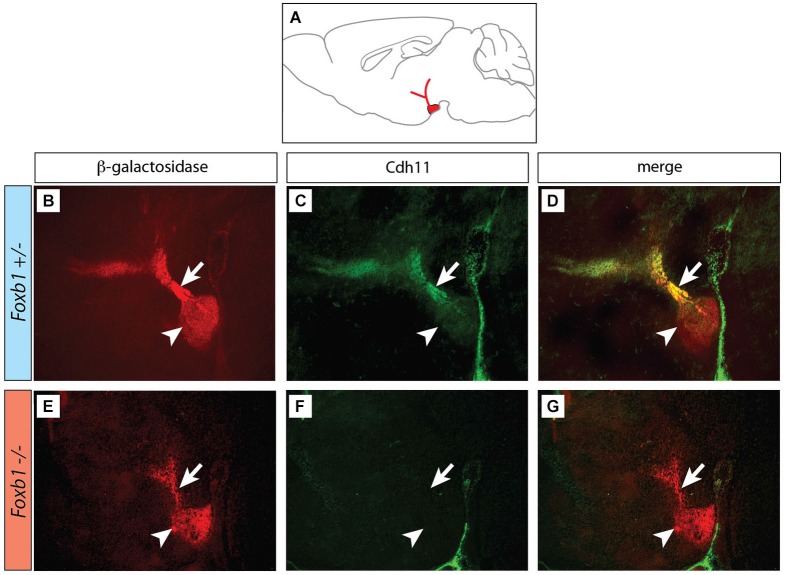
**Decreased immunocytochemical visualization of Cdh11 in the *Foxb1* mutant MBO. (A)** Position of the MBO on a sagittal diagram of the mouse brain, rostral to the left. **(B–G)** Sagittal sections (rostral to the left) of *Foxb1 +/−* **(B–D)** and *Foxb1* −/− **(E–G)** E18.5 MBO. Antibody against reporter protein β-galactosidase **(B,E)** labels MBO (arrowheads) and mammillary axonal tree (arrows) in both* Foxb1 +/−* **(B)** and *Foxb1* −/−**(E)**. Antibody against Cdh11 labels MBO (arrowhead) and axonal bundle (arrow) in the *Foxb1 +/−* **(C,D)** but not in the *Foxb1* −/− **(F,G)**.

**Figure 3:**
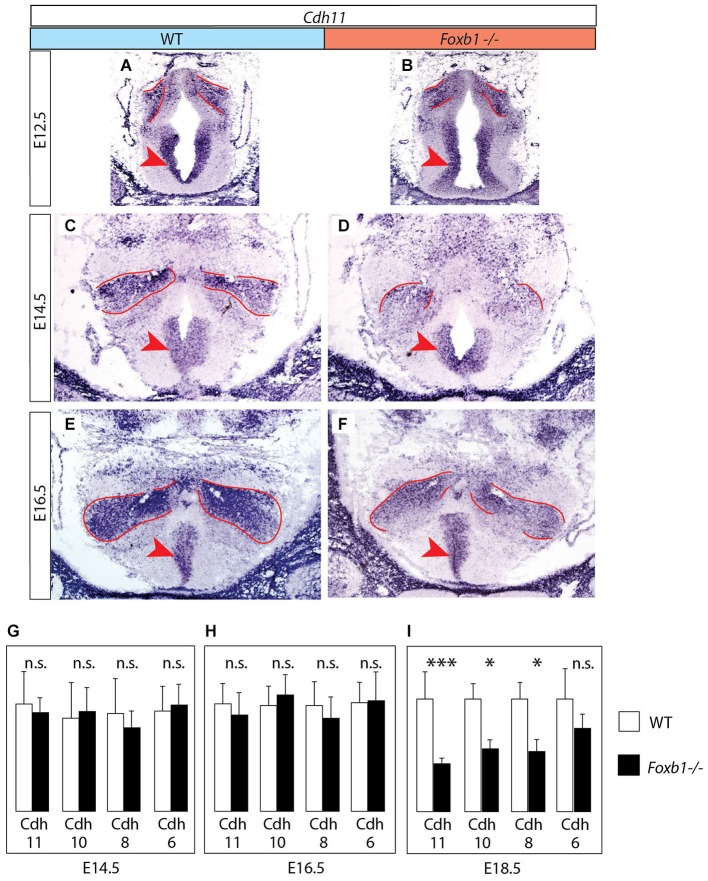
**Decrease in developmental *Cdh11* expression in the *Foxb1* mutant MBO. (A–F)** *Cdh11 in situ* hybridization on transverse sections of the MBO in wild type **(A,C,E)** and *Foxb1* homozygous embryos **(B,D,F)** at E12.5 **(A,B)**, E14.5 **(C,D)** and E16.5 **(E,F)**. The approximate boundaries of the MBO have been outlined in red. **(G–I)** Quantitative RT-PCR for *Cdh6*, *8*, *10* and *11* in the mammillary region (embryonic ages and genotypes as indicated). Significant differences are detected only at E18.5 by this method, probably due to the presence of *Cdh11* expression in areas outside the MBO (arrowheads in **(A–F)**) not affected by the *Foxb1* mutation. Mean ± SD; * = *P* < 0.05; *** = *P* < 0.001; n.s. = not significant.

**Figure 4:**
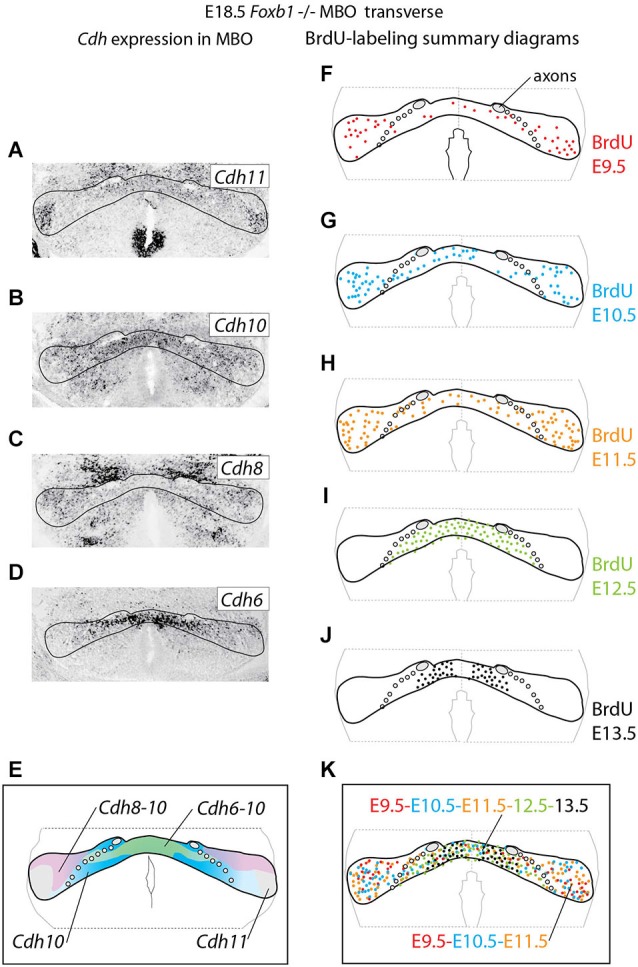
**Cadherin expression alteration and cell sorting phenotype in the *Foxb1* −/− MBO. (A–D)** ISH for Cadherins (as indicated) on transverse sections of *Foxb1 −/− MBO* at E18.5. **(E)** Diagram showing the combined expression patterns of the Cadherins investigated on a schematic of the *Foxb1* −/− MBO. **(F–J)** Diagrams of BrdU-labeled cells after injections from E9.5 to E13.5 as indicated **(K)** Diagram of neurogenetic dates in the *Foxb1* −/− MBO after the data in **(F–J)**.

**Figure 5:**
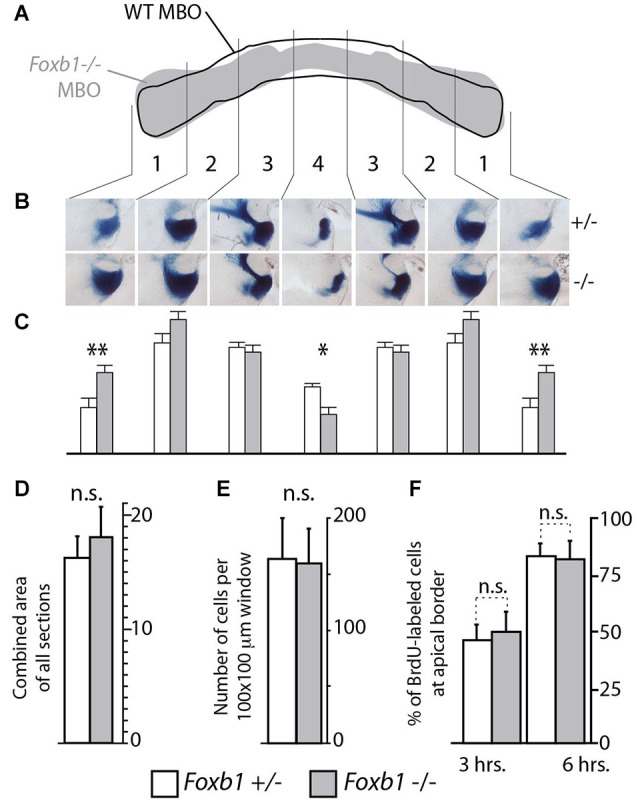
**Shape, but not size, altered in the *Foxb1* −/− MBO. (A)** Superimposing the outline of the labeled area in transverse section of the wild type and *Foxb1* −/− MBO suggests a change in morphology in the mutant. **(B)** Thick sagittal sections of *Foxb1* +/− and*−/−* labeled with X-gal reaction and compared according to 4 arbitrary latero-medial regions of equal size (numbered 1–4) confirm this impression (in heterozygotes as well as homozygotes, only one allele expressed β-galactosidase, see Methods section for details)**. (C)** The combined stained area of the sections in **(B)**, for each of the 4 latero-medial regions shows significantly increased lateral size and significantly reduced medial size for the *Foxb1* −/− MBO**. (D)** No significant differences in total volume of the MBO between *Foxb1* +/− and *Foxb1* −/− (combined stained area of all sections). **(E)** No significant differences in cell density difference between *Foxb1* +/− and *Foxb1* −/−**. (F)** No significant difference in proliferation in the mammillary neuroepithelium between *Foxb1* +/− and *Foxb1* −/−. The cells were counted either 3 h (left) or 6 h (right) after BrdU injection at E12.5. **(D–F)** One-way ANOVA; mean ± SD; n.s., not significant.

**Figure 6:**
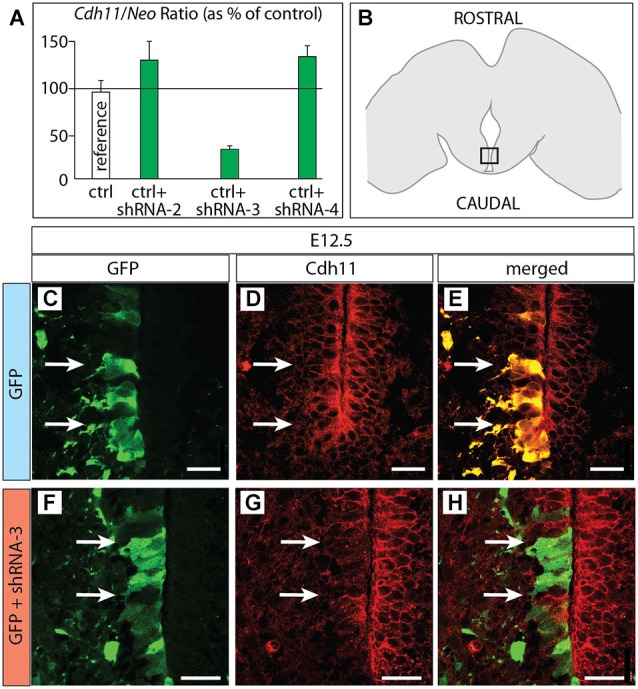
**Cdh11 protein decreased after *in utero* RNAi. (A)** Percent of *Cdh11* knockdown relative to *Neo* knockdown after transfection of different shRNA plasmids against *Cdh11* into HEK239 cells in culture (see Materials and Methods for details). **(B)** The frame indicates the localization of the photographs shown in **(C–H)** on a horizontal brain section. **(C–H)** Horizontal sections of E18.5 of control **(C–E)** and *Cdh11* knockdown **(F–H)** MBO neuroepithelium. The position of cells labeled by GFP is shown by two arrows. Scale bar 50 μm.

**Figure 7:**
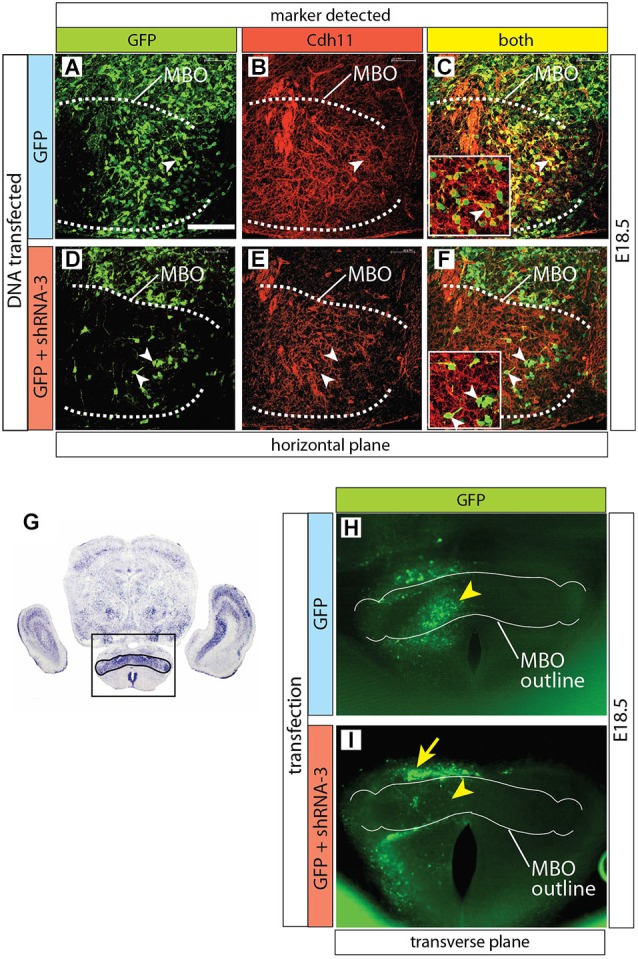
**Cdh11 protein decreased in MBO cells after *Cdh11*-knockdown. (A–F)** Horizontal sections of E16.5 left side MBO (brain midline to the right of each photo), labeled with anti-Cdh11 antibody after *in utero* electroporation with control plasmid alone **(A–C)** or together with shRNA-3 **(D–F)** at E12.5. A dotted line delimits *Cdh11*-expressing left MBO. Arrowheads in D-F show GFP-expressing, non-*Cdh11*-expressing cells**. (G)** Transverse section of E18.5 brain showing Cdh11 detection by ISH. The frame includes the MBO (identified by strong Cdh11 expression) and indicates the approximate area of the image in **(H,I). (H,I)** Transverse vibratome sections of wild type E18.5 MBO after electroporation of plasmids containing GFP reporter alone **(H)** or together with shRNA-3 plasmid **(I)**, with an added outline of the MBO for reference. Arrowheads indicate equivalent positions in the control **(H)** and knockdown MBO **(I).** The arrow in **(I)** indicates an abnormal band of labeled cells outside the knockdown MBO. Scale bar (in **A**) 100 μm.

**Figure 8:**
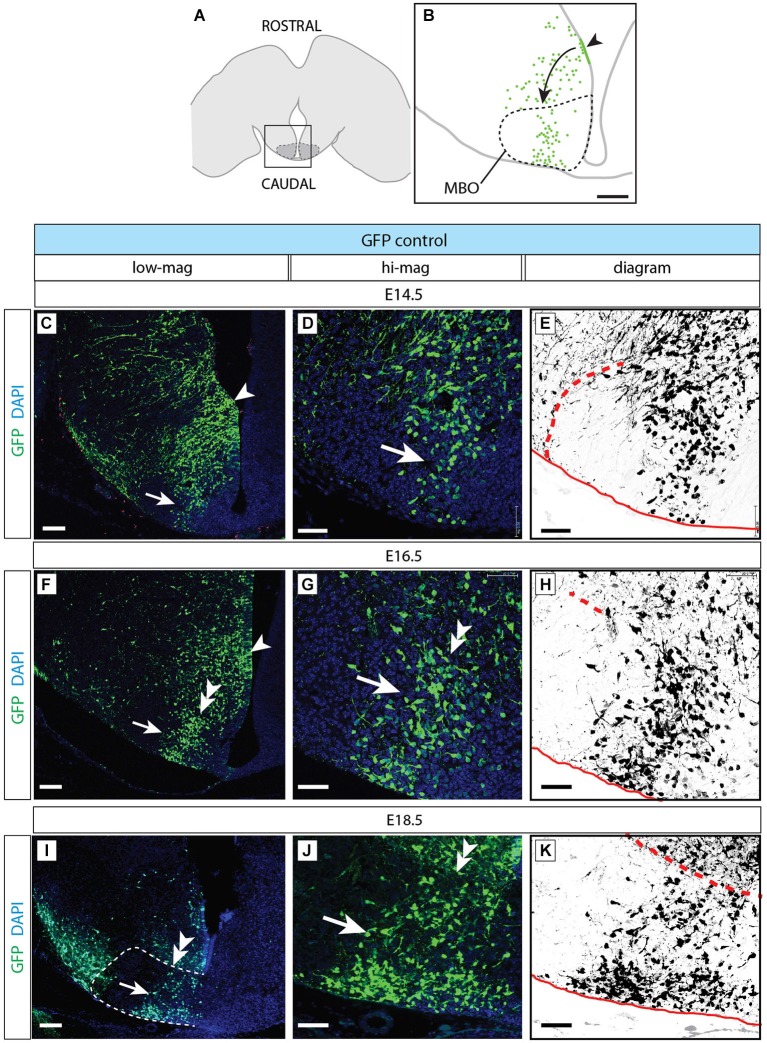
**Normal development of MBO after GFP transfection. (A)** The frame indicates the MBO (dotted line) and the approximate localization of the photographs shown in **(C,F,I)** on a horizontal brain section. **(B)** The region framed in **(A)** showing the MBO, the transfected neuroepithelium (arrowhead) and the GFP-labeled migrating cells (arrow) entering the MBO in a control animal at E18.5. **(C–K)** Control-transfected brains (orientation as in **B**), ages as indicated. Every series has a low-magnification image (left), a high-magnification view of the previous (center) and a diagram for clarity (right). Arrowheads indicate the labeled neuroepithelium; arrows show labeled cells in the MBO; double arrowheads mark the rostral MBO boundary. Scale bar in **(B,C,F,I)**, 100 μm; in **(D,E,G,H,J,K)**, 50 μm.

**Figure 9:**
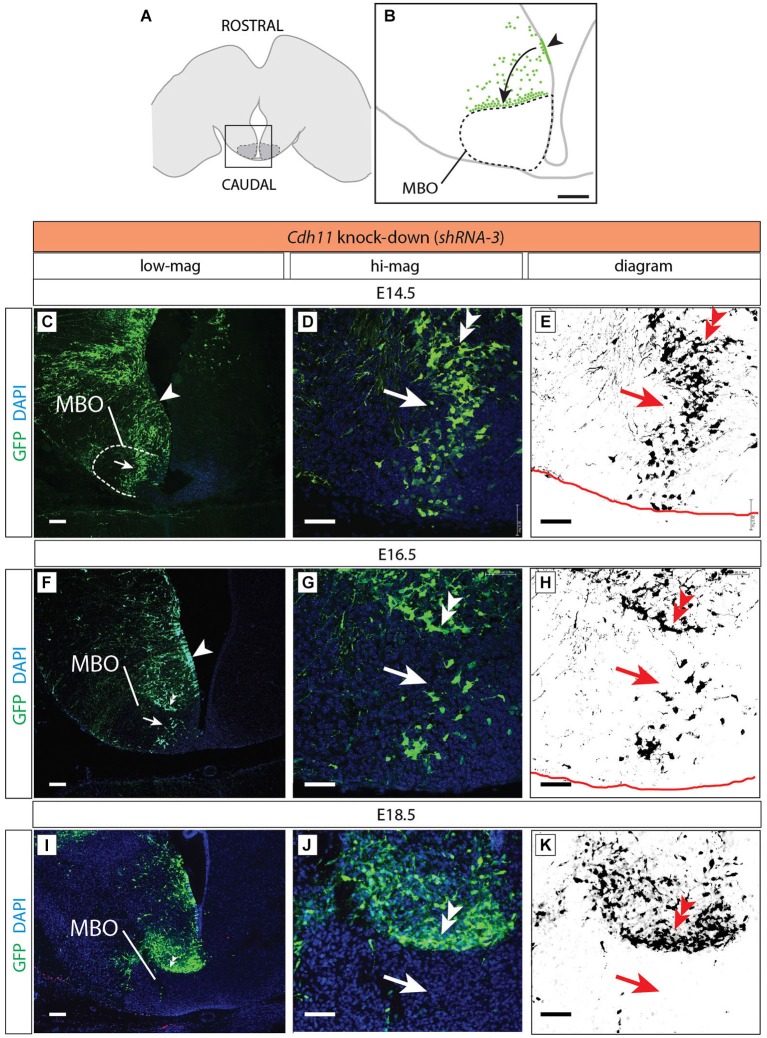
***Cdh11*-knockdown neurons accumulate outside the MBO. (A)** The frame indicates the MBO (dotted line) and the approximate localization of the photographs shown in **(C)**, **(F)** and **(I)** on a horizontal brain section. **(B)** The region framed in **(A)** showing the MBO, the transfected neuroepithelium (arrowhead) and the GFP-labeled migrating cells (arrow) accumulating at the boundary of the MBO in a *Cdh11*-knockdown animal at E18.5. **(C–K)** *Cdh11*-knockdown-transfected brains (orientation as in **B**), ages as indicated. Images labeled as in Figure 5. Scale bar in **(B,C,F,I)**, 100 μm, in **(D,E,G,H,J,K)**, 50 μm.

**Figure 10:**
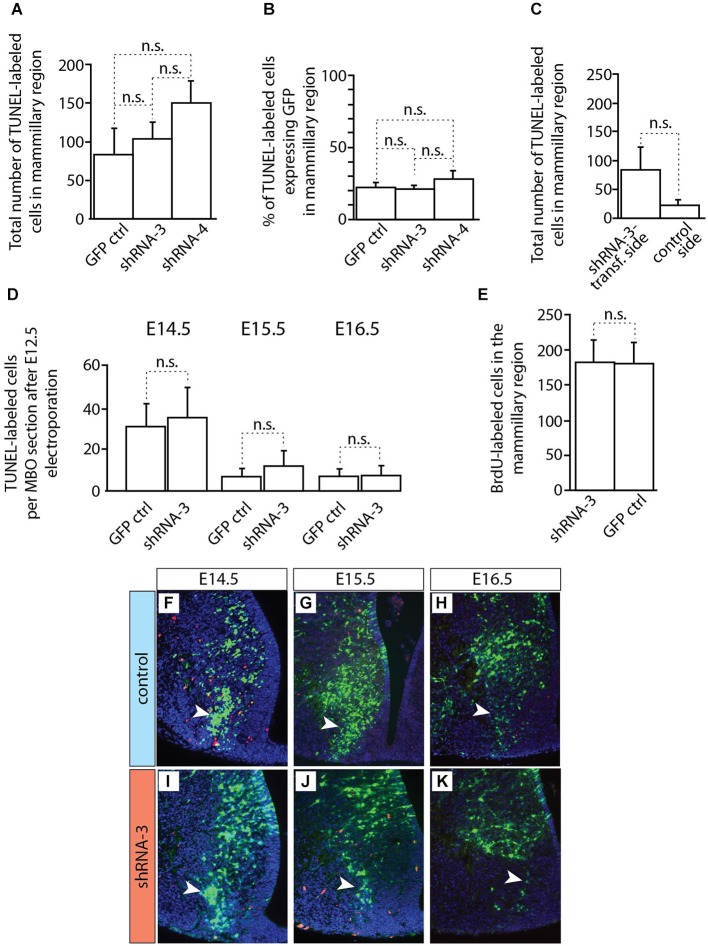
**Apoptosis and proliferation not changed in MBO after *in utero* electroporation. (A–D)** Countings of TUNEL-labeled (apoptotic) cells on horizontal sections of brains transfected by *in utero* electroporation at E12.5 with either control or knockdown constructs (as indicated). The age of data collection is E18.5 except for **(D)** which shows three earlier ages (as indicated). Mean ± SD; n.s. = not significant. **(E)** Countings of BrdU-labeled cells on horizontal sections (E18.5) of the mammillary region after transfection at E12.5 of experimental or control constructs (as indicated). The BrdU was injected at the time of electroporation. Mean ± SD; n.s. = not significant. **(F–K)** TUNEL staining on the electroporated side of the mammillary region of E18.5 brains transfected with control **(F–H)** or knockdown **(I–K)** plasmids. Red cells: TUNEL-labeled (apoptosis). Arrowheads indicate the labeled cells inside the MBO.

**Figure 11:**
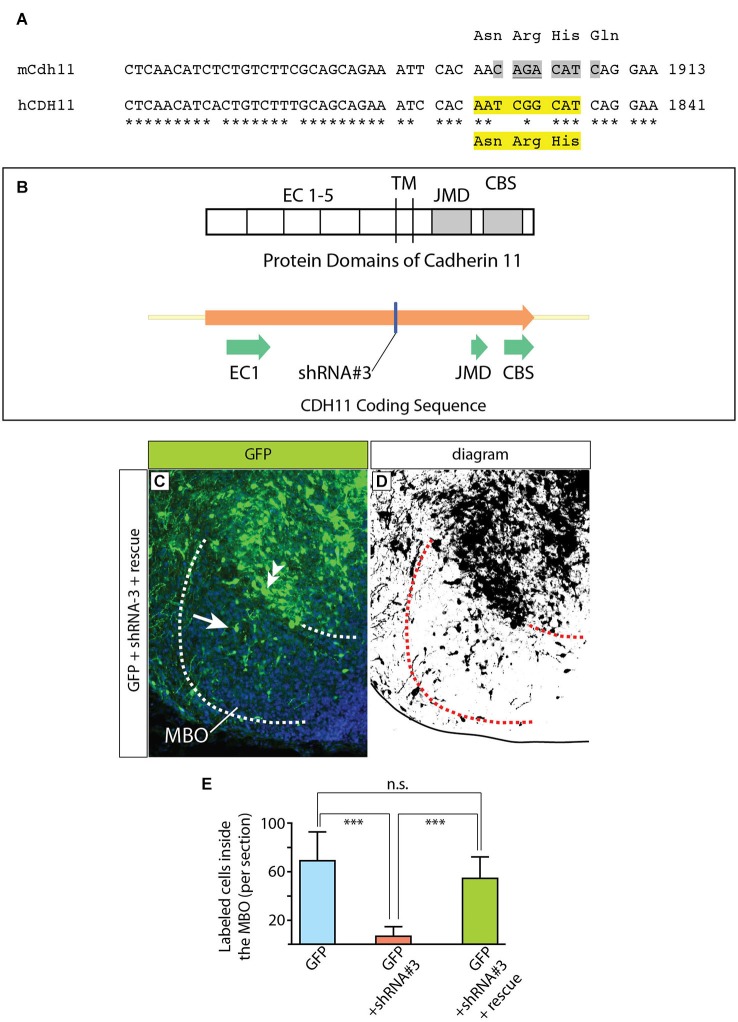
**Complementation construct and rescue experiment. (A)** Mouse/human *Cdh11* sequence comparison of a small stretch corresponding to EC5 and including the “seed region” for shRNA-3. The asterisks indicate mouse/human-identical nucleotides; the gray highlights in mCdh11 show the shRNA-3 “seed region”; the yellow highlights in hCDH11 indicate the deleted nucleotides (AAT CGG CAT) and the corresponding missing amino acids (Asn-Arg-His) in the resulting human Cadherin11 protein. **(B)** The missing three aminoacids are part of the juxtamembrane domain (JMD) of the Cadherin 11 protein. **(C)** Horizontal section of the left side of the E18.5 MBO (brain midline to the right side of the photo) after electroporation at E12.5 with a mixture of plasmids encoding GFP (reporter) shRNA#3 (Cdh11-knockdown) and rescue construct (see Materials and Methods for details). **(D)** High-contrast version of the photograph in **(C)**. **(E)** GFP-labeled cell countings on horizontal sections of E18.5 MBO after transfection at E12.5 with GFP reporter plasmid (blue), GFP plus shRNA#3 (red) and GFP plus shRNA#3 plasmid plus rescue plasmid (green).

To make this CDH11 immune to RNAi by the shRNA-3, the “seed sequence” (Lai, 2002; Lewis et al., 2003), required for target recognition by the shRNA-3 and subsequent degradation was deleted (Figures 11A,B). The deleted seed sequence encodes three amino acids in the extracellular domain EC5 in principle not involved in the adhesive or signaling function (Leckband and Prakasam, 2006; Ciatto et al., 2010; Harrison et al., 2010) of cadherins. The complementation construct was mixed with shRNA-3 construct (1:1) and then transfected into the developing MBO by *in utero* electroporation. The results were analyzed as before.

### Birthdate Analysis in the Wild Type and *Foxb1* −/− MBO

Pregnant mice were intraperitoneally injected with bromodeoxyuridine (BrdU) (RPN201; GE Healthcare) (50 μg/g body weight) at the appropriate gestational age (from E9.5 to E13.5). The injections took place at 12:00 P.M., 3:00 P.M., and 6:00 P.M. (Takahashi et al., 1993) and the fetuses were collected at E18.5. We detected cell proliferation on cryosections (20 μm) by means of anti-BrdU antibody M0744 (1:100) (Dako), after epitope retrieval in 2 M HCl for 30 min at 37°C.

#### *In Situ* Hybridization (ISH) on Sections

Nonradioactive ISH was performed on cryosections (20 μm thick) that were fixed in 4% paraformaldehyde and acetylated after sectioning. Prehybridization, hybridization, and washing steps were performed with the help of an automatic liquid-handling unit (Genesis RSP 200; Tecan), and the digoxigenin-labeled probe was detected by a dual-amplification procedure.

#### Quantitative Real-Time PCR

The posterior ventral part of the hypothalamus of *Foxb1* −/− and wild type animals was dissected, the tissue was homogenized and mRNA extracted with the Dynabeads mRNA DIRECT kit (Invitrogen). Reverse Transcription was performed with the Transcriptor First Strand cDNA Synthesis kit (Roche) using anchored-oligo(dT) and random hexamer primers. The cDNA was amplified in a Bio-Rad iCycler using SYBR Green Supermix (Bio-Rad) and the following gene-specific primers:

- Cdh11: forward primer: 5′GGACGACACAGCCAATGGACCAAG 3′, reverse primer: 5′CTCCACGTCGGGCATATACTCCTG 3′;
- Cdh6: forward primer: 5′AGCAAAGCAGCCGCGTTCCTCT 3′, reverse primer: 5′TCATCCTTGTCAACAGCACGCAGG 3′;
- Cdh8: forward primer: 5′ ACAAAGACGATCCCAAAAACGGAC 3′, reverse primer: 5′CATTATGTTTTGCCAGAATGCTCA 3′;
- Cdh10: forward primer: 5′CTCGTGTGTCTGTTTTTGTGAGGA 3′, reverse primer: 5′ TTCGGATTCACAGCAGCCAAACTG 3′.

The PCR was performed in triplicates for each sample with three samples per genotype and normalized to house-keeping gene EF1 alpha as control.

#### Apoptosis Detection

We sectioned (20 μm thickness) with a cryostat E14.5, E15.5 and E16.5 brains electroporated at E12.5. We selected the sections containing the MBO, pretreated them with proteinase K (1.5 μg/ml, 5 min) at room temperature and labeled the apoptotic cells with the ApopTag TUNEL (terminal deoxynucleotidyl transferase-mediated biotinylated UTP nick end labeling) kit (Millipore Bioscience Research Reagents) according to the instructions of the manufacturer. We used DAPI as counterstain and counted the absolute number of apoptotic cells in the posterior ventral part of the hypothalamus under 20x magnification in three histological sections per animal and in three individuals per treatment.

#### Proliferation After *In Utero* Electroporation of shRNA-3

Mouse embryos were transfected by *in utero* electroporation with *GFP*-control plasmid alone or together with shRNA-3 at E12.5, received BrdU at E13.5 (through intraperitoneal injection of the pregnant dam) (see above, Neuronal birthdate analysis) and their brains were collected at E14.5. Three control and three experimental embryonic brains were analyzed. For each of them, five horizontal sections (20 μm thick) through the MBO were treated with anti-BrdU and anti-GFP antibodies and examined under the confocal microscope. We counted BrdU-labeled cells in 100 μm × 200 μm bins covering the width of the neuroepithelium in the GFP-positive area of the neuroepithelium of the mammillary recess next to the MBO.

#### Proliferation in the *Foxb1* Mutant

We injected pregnant dams intraperitoneally with BrdU at E12.5 and collected the embryos for analysis either 3 h or 6 h later. Three embryos of each genotype (homozygotes vs. and heterozygotes) were analyzed. For each of them, seven to twelve horizontal sections (12 μm thick) through the MBO were reacted with anti-GFP antibody to identify the mammillary neuroepithelium (in this mutant, expression of reporter gene *GFP* is a proxy for *Foxb1* transcriptional activation) as well as with anti-BrdU antibody and nuclear marker DAPI. We counted all cells on the apical border of the *GFP*-expressing mammillary neuroepithelium and scored them as BrdU-labeled or unlabeled.

#### Statistical Analysis

We used Prism 6 software (GraphPad Software Inc., La Jolla, California) to calculate the one-way ANOVA. The results are represented as mean ± Standard Deviation (SD).

## Results

### Neuronal Birthdate Pattern does not Match *Cdh* Expression Pattern in the MBO

We hypothesized a simple mechanism to build the MBO. Since neurons fated for a certain specific MBO subnucleus are born during the same wave of neurogenesis (neurogenetic paradigm, Altman and Bayer, 1988; Bayer and Altman, 1995a), these neurons would then express the same cadherin combination and so they would aggregate together. This hypothesis predicts that the patterns of birthdating and cadherin expression combinations should match each other. That is, an MBO subnucleus would be born at a specific time and express a specific cadherin combination. This would be a direct and immediate way to prove that cadherin combinations underlie brain architecture.

We chose E18.5 as the age of analysis, since at this age all MBO neurons have been born, have completed migration and have settled in their final position; in addition, cadherin expression in the MBO gradually decreases and becomes less patterned after birth and through the adult stage (data not shown).

We first used ISH to label expression of the four classical cadherins of Type II present in the developing MBO (Kimura et al., 1996; Suzuki et al., 1997) at E18.5 (Figures 1A–F). We then labeled embryonic brains with proliferation marker BrdU at E9.5 through E13.5 and mapped the labeled cells at E18.5 (Figures 1G–L). We found that, on transverse sections at this age, MBO neurons are arranged in bands or strata (Figure 1L) according to an “outside-in” model. The neurons born first (E9.5) settled most laterally (“outside”) and younger neurons would settle gradually more medial, with the last born (E13.5) in the medialmost position by the third ventricle. Additionally, analysis on sagittal sections (not shown) indicated an anterior-lateral-dorsal (early born) to posterior-medial-ventral (late born) gradient, consistent with classical descriptions (Altman and Bayer, 1986). The outside-in chronological arrangement matches as well the latero-medial partition of the MBO into histological subnuclei (Allen and Hopkins, 1988).

Comparison of the two data sets revealed that the combinatorial domains of cadherin expression do not match the birthdating bands revealed by BrdU (Figures 1F,L). Instead, each MBO neuron seems to belong at the same time to two different, intersecting groups, one of them determined by birthdate and the other by cadherin combination. Therefore, the chronological arrangement of MBO subnuclei cannot be due to cadherin expression combinations. Intriguingly, however, one characteristic was common to the entire MBO, and this was the intense expression of *Cdh11* (Figure 1B; Allen and Hopkins, 1988). We hypothesized that this one cadherin could somehow be the “universal glue” keeping together the two intersecting systems of the MBO.

### *Cdh11* Expression in the MBO is Maintained by Transcription Factor *Foxb1*

Next we looked for ways to study MBO architecture in conditions of reduced *Cdh11* expression. *Foxb1* is a transcription factor gene specifically expressed in the developing MBO (Kaestner et al., 1996; Alvarez-Bolado et al., 2000b) and essential for the development of the mammillary axons (Alvarez-Bolado et al., 2000a; Kloetzli et al., 2001; Szabó et al., 2011). Since *Cdh11* has been implicated in axonal development and circuit formation (Marthiens et al., 2005; Paradis et al., 2007; Ross et al., 2012), we asked if *Foxb1* could be involved in the regulation of *Cdh11* expression in the MBO. Cdh11 protein was absent from the *Foxb1* −/− MBO at E18.5 (Figure 2). Since we can detect *Cdh11* mRNA in the mutant MBO at E12.5, E14.5 and E16.5 by ISH (Figures 3A–F) as well as quantitative RT-PCR (Figures 3G–I), but we cannot detect it anymore at E18.5 (Figure 2), we assume that *Foxb1* is necessary not for activating *Cdh11* expression in the MBO but only for its maintenance. This is a previously unreported role of transcription factor Foxb1 in the development of this part of the hypothalamus. The residual expression of *Cdh11* in the MBO at E18.5 by quantitative RT-PCR (Figure 3I) is probably due to a periventricular layer (outside the MBO) which does not change in the mutant (arrowheads in Figures 3A–F). Additionally, *Cdh6* and *Cdh8* showed a slight reduction in expression after E16.5 in the *Foxb1* mutant (Figure 3I).

We concluded that* Foxb1* is required for maintenance of *Cdh11* expression in the developing MBO, adding to the list of forkhead-regulated cadherin genes like *E-cadherin* (*Cdh1)* (Cha et al., 2007), *Cdh3* (Habashy et al., 2008), *Cdh5* (Kalinichenko et al., 2002) and *Cdh7* (Dottori et al., 2001).

### Decrease in *Cdh11* Expression in the Entire MBO Alters Cell Sorting

Next we wanted to use the *Foxb1* mutant in order to test the hypothesis that intense expression of *Cdh11* could be acting as a general glue, overriding any in principle possible effect of the cadherin combinations. Therefore we analyzed cadherin expression and birthdate of the different cell populations in the *Foxb1* mutant MBO at E18.5 (Figure 4). The strong decrease in *Cdh11* expression in the MBO at E18.5 (Figure 2F) was confirmed by ISH (Figure 4A; compare with Figure 1B for a control). The domains of expression of *Cdh10*, *8* and *6* in the mutant MBO were rearranged (Figures 4B–D, summarized in E; compare to Figures 1A–F). Comparison of the distribution of neuronal birthdates in the mutant (Figures 4F–K) and in the wild type MBO (Figures 1G–L) was very informative. In the mutant, neurons sharing a birthdate were spread over a large area, did not form separate bands, and were mixed with neurons of different birthdate (Figure 4K). We concluded that the late, gradual loss of *Cdh11* expression in the entire developing MBO leads to disruption of the chronological arrangement of the MBO neurons.

### Decrease in *Cdh11* Expression Alters Morphology but not Size of the MBO

Since cell sorting is an important mechanism underlying the development of a typical, characteristic shape of the different organs (reviewed in (Lecuit and Lenne, 2007)), we expected the overall morphology of the *Foxb1* mutant MBO to change. To confirm this prediction, we took advantage of the two existing null mutant alleles of *Foxb1*, which carry different reporters. The *Foxb1-tauLacZ* (Alvarez-Bolado et al., 2000a) produces beta-galactosidase as reporter, while expression of the *Foxb1-Cre* allele is reported by EGFP (Zhao et al., 2007). By crossing these mutants, we generated *Foxb1* heterozygous mutants carrying one allele expressing beta-galactosidase (and a wild type one of course), and homozygous mutants carrying also only one allele expressing beta-galactosidase (and another expressing EGFP). In this way, the amount of beta-galactosidase expressed per cell is the same in heterozygotes and homozygotes, and as a consequence we can use beta-galactosidase as a marker for comparison (we discarded the heterozygotes expressing GFP). We know that *Foxb1* is expressed in the entire MBO (Alvarez-Bolado et al., 2000b), and therefore, expression of beta-galactosidase is a good reporter of MBO morphology and size. Observation of transverse sections of the MBO of both genotypes labeled with antibody against beta-galactosidase showed a change in MBO morphology in the mutant (diagram in Figure 5A). Measuring the size of every one of four mediolateral regions of the MBO (see Materials and Methods section) revealed significant reduction in the most medial region and significant enlargement in the most lateral region of the homozygous MBO (Figures 5B,C). Remarkably, the overall size of the mutant MBO was not different from that of the heterozygous (Figure 5D). To further support this claim, we ascertained that there is no difference in cell density (Figure 5E) or in proliferation (Figure 5F) in the mutant MBO. As expected after an alteration of cell sorting, the MBO morphology was affected while its overall size remained unaffected.

### Knocking Down *Cdh11* by RNA Interference

Based on the hypothesis that *Cdh11* expression keeps the chronological arrangement of MBO neurons, we then predicted that MBO-fated migrating neurons lacking Cdh11 would fail to enter a wild-type,* Cdh11*-expressing MBO. To test the prediction, we decided to use RNA interference (RNAi; Paddison et al., 2002) *in utero* in order to reduce *Cdh11* expression in MBO neurons born at a specific time point, then analyze their position several days later. We tested different commercially obtained plasmid-encoded small hairpin (sh)RNA against *Cdh11* in culture (see Methods section for details) and found that transfection of shRNA-3 resulted in powerful knockdown of *Cdh11* in culture (Figure 6A). We then used *in utero* electroporation to transfect shRNA-3 into the neuroepithelium lining the mammillary recess of the third ventricle, where MBO neurons are born (Figure 6B). We chose E12.5 as time point for the experiment, since at this age the MBO is accessible to DNA transfection through *in utero* electroporation (Haddad-Tóvolli et al., 2013). Transfection of GFP-expressing reporter plasmid at E12.5 into the mammillary recess resulted in an abundance of labeled neuroepithelial cells as can be seen in horizontal sections (Figure 6C). Cdh11 could be detected with antibodies in the same cells (Figures 6D,E). A very different picture could be seen when *Cdh11* mRNA was knocked down in the neuroepithelium. Although numerous neuroepithelial cells were labeled with GFP (Figure 6F), Cdh11 protein could not be detected in them (Figures 6G,H).

Furthermore, Cdh11 protein could not be detected in MBO neurons of *Cdh11*-knockdown brains either (Figures 7A–F). Finally, by screening for GFP expression on transverse Vibratome sections of transfected MBO we detected a clear and consistent pattern alteration after transfection with shRNA-3 (Figures 7G–I).

In conclusion, at this point we were able to specifically knockdown *Cdh11* expression in culture and in the MBO developing *in utero*.

### Control-Transfected MBO-Fated Neurons Form a Defined Group Inside the MBO

We then used antibody detection of GFP on horizontal sections in order to analyze the position of control-transfected neurons at different time points (Figure 8). Transfection was performed at E12.5 (Figures 8A,B). Two days after transfection, a number of GFP-labeled neurons was present in the MBO forming a well-defined stream extending from rostral to caudal through the MBO (Figures 8C–E). These neurons were placed at the most medial side of the MBO, as expected following the general “outside-in” settling pattern typical of the hypothalamus. The latest arrived neurons appose themselves to earlier populations from the medial side, so that the oldest neurons (born at E9.5) will end up forming the most lateral (“outside”) part of the nucleus and the youngest (born at E13.5) the most medial (Figures 1G–L). As expected, neurons born before transfection age (E12.5) had arrived earlier to the MBO, occupied more lateral positions and were unlabeled (Figures 8C–E).

Labeled cells arrived to the MBO through E16.5 (Figures 8F–H) and E18.5 (Figures 8I–K) and they remained recognizable as a stable, compact group on the lateral side of the nucleus.

These results show that we can use *in utero* transfection to label neurons born at a certain age and that these neurons form an identifiable group consistently entering the MBO as a stream and consistently settling in a position corresponding to their birthdate.

### *Cdh11*-Knockdown Neurons Accumulate Outside the MBO

*Cdh11*-knockdown transfected neurons behaved in a quite different way (Figure 9). Already at E14.5 the stream of labeled cells did not form a straight, rostro-caudally oriented group but seemed deformed in the medial direction, towards the midline (arrow in Figures 9C–E). At the same time, labeled cells started to abnormally accumulate on the rostral side of the MBO (double arrowhead in Figures 9D,E). Two days later (E16.5), only few labeled cells were still to be found in the MBO (arrow in Figure 9G). The labeled cells abnormally gathering rostral to the MBO formed an elongated, medio-laterally oriented aggregate (double arrowhead in Figures 9G,H). At E18.5 there were virtually no labeled cells in the MBO. The rostral border of this nucleus however was easy to recognize because of a large accumulation of labeled cells (double arrowhead in Figures 9I–K). We checked for apoptosis and proliferation effects in order to discard these phenomena as causes of the decrease in labeled cells in the MBO (Figure 10).

The definitive control for an RNAi experiment is the rescue by expression of a form of the target gene resistant to siRNA (Anonymous-Editorial, 2003). Accordingly, we performed rescue experiments based on co-transfection of DNA constructs expressing non-interferable *Cdh11* (see Material and Methods for details). These experiments consist of introducing in the cells a form of Cdh11 that has been mutated in such a way that it preserves its adhesive domains while losing the domain that is recognized by the shRNA (Figures 11A,B). We would expect that cells transfected in this way would show a lesser effect of the shRNA interference, since shRNA will be able to degrade endogenous Cdh11, but not the “non-interferable” Cdh11 that we are cotransfecting. The results of these experiments (Figures 11C–E) show many more GFP-labeled cells inside the MBO in “rescued” animals than in animals treated only with shRNA-3. In this way, we confirmed the specificity of our previous RNAi experiments. We concluded that experimental reduction of *Cdh11* expression in one specific MBO neuronal subpopulation during development causes that subpopulation to accumulate outside the MBO.

## Discussion

Three insights have been considered as key to understand cell sorting in brain development—the importance of information encoded in neuronal birthdates (Bayer and Altman, 1987) and in cadherin combinations (Suzuki et al., 1997; Price et al., 2002), and the importance of non-specific adhesion phenomena (Foty et al., 1996). In this work we combine for the first time these insights by showing: (1) that one-cadherin adhesion has the power to organize the neurons of a brain nucleus according to dates of neurogenesis; and (2) that cadherin combinations and one-cadherin-adhesion have different roles and different mechanisms working sequentially to fulfill different roles through different mechanisms.

In the developing MBO, two sources of information, i.e., neuronal birthdates and cadherin combinations, work to secure appropriate connections between MBO neurons and their anterior thalamic targets. Neuronal birthdates ensure appropriate medio-lateral correspondence between MBO subdivisions and the anterior thalamic nuclei that are their specific targets. Cadherin combinations presumably take care of the last step in navigation, identifying the individual target neurons inside the thalamus.

These two sources of information are maintained through a hierarchy of adhesions. First, Cdh11 allows entrance of the successively arriving neurons into the target nucleus, then, again Cdh11 keeps them organized chronologically. Cdh11 prevents also weaker, combination-based adhesion forces from intermixing the birthdate-based organization. Finally, the cadherin combinations would underlie appropriate fasciculation of axons projecting to same area within a target region (Wöhrn et al., 1999; Treubert-Zimmermann et al., 2002), and could be responsible for the final identification of the target neurons as well.

### Cdh11, One Cadherin to Rule Them All

Our first finding, that birthdates and cadherin combinations do not coincide in the MBO (Figure 1) (reminiscent of similar results in the avian and mouse striatum (Redies et al., 2002; Heyers et al., 2003)) is surprising. How could neurons born on a certain date aggregate together (Bayer and Altman, 1995b) if not by expressing specific combinations of adhesive molecules (Redies and Puelles, 2001). A possible answer can be found in Figures 4, 5, which show that abolition of *Cdh11* expression (*Foxb1* mutant) alters sorting in the MBO, causing a mixing of the combinatorial groups as well as the birthdate groups. These results suggests that intense, generalized Cdh11-based adhesion would make all MBO neurons homogeneously highly adhesive overriding the effect of the subordinate interactions based on the combinations of* Cdh6, 8* and *10* otherwise present in MBO neurons. In this way, Cdh11 would ensure that the newly arrived neurons appose themselves from the medial side to the previously arrived (“outside-in” arrangement) rather than mixing with each other based on weaker variegated interactions based on the combinations. The results of the knockdown experiments (Figures 6–9) reinforce this insight by showing that, without *Cdh11* expression, newly arrived neurons are excluded from the MBO. This is reminiscent of the DAH prediction that less adhesive cells will remain on the periphery (Steinberg, 1963). MBO neurons keep a medio-lateral correspondence with their targets in the anterior thalamus—the most medial MBO neurons project to the most medial anterior thalamic neurons, and the most lateral to the most lateral (Seki and Zyo, 1984). We suggest that the function of Chd11-based adhesion is to keep the MBO subdivisions approximately in register with their targets in the ATC, making sure that their axons enter the target region in the appropriate neighborhood. Accordingly, when the developing mammillothalamic axonal tract reaches the anterior thalamus, its axons separate into three bundles which innervate their targets sequentially from medial to lateral (Alpeeva and Makarenko, 2009).

### Role of the Cadherin Combinations

What would then be the role of the cadherin combinations? We propose that, in the MBO, combinatorial adhesion adds one further layer of specificity to the connections between MBO and anterior thalamus. After appropriate, medio-laterally organized entrance of MBO axons in their target region, the anterior thalamus, and since anterior thalamic neurons express combinations of *Cdh6*, *8* and *10* (Suzuki et al., 1997; Bekirov et al., 2002) matching those expressed by the incoming MBO axons, these can rely on the combinatorial code for the final target identification. In this way, the two intersecting, cadherin-based sorting systems of the MBO guarantee appropriate neighborhood targeting (Cdh11) and fine-grained “address” targeting (combinations). *Cdh6*, *8*, *10* and *11* have all been shown indispensable for appropriate synaptic connectivity in a variety of systems (Suzuki et al., 1997; Paradis et al., 2007; Osterhout et al., 2011; Williams et al., 2011; Ross et al., 2012). Cadherin-dependent specific fasciculation, experimentally demonstrated in other systems (Treubert-Zimmermann et al., 2002) could play a role also here. Appropriate connectivity based on birthdates has been suggested as a general principle in brain development (Bayer and Altman, 1995b).

### A Hierarchy of Homotypic Interactions

Incidentally, the proposed role of *Cdh11* as “central clasp” can be understood as non-specific, that is, based on stronger adhesion, not on combinations (despite being homophilic, i.e., Cdh11-Cdh11). This means that Cdh11 is not part of the combinations but overrides them all. On this basis we can predict that, when expression of *Cdh11* decreases (i.e., in the *Foxb1* mutant), MBO neurons recover their underlying differential adhesivities, which are due to differential expression intensity of various adhesion molecules other than *Cdh11*, and reorganize accordingly. A key assumption for this interpretation is that the combinations provide weaker adhesion than the homogeneous expression of *Cdh11*. This conjecture is borne out by the phenotype. In addition, the appearance of the *Cdh11*-knockdown neurons gathered at the boundary of the MBO (Figures 8, 9) brings to mind the DAH prediction that the least adhesive cells will remain on the surface of a more adhesive “bulk” (Steinberg, 1963). Perhaps the dicotomies “homophilic vs. heterophilic” and “specific vs. non-specific” should be substituted by more flexible concepts.

### Caveats

*Cdh11*-knockdown neurons could simply be migration-impaired, since cadherins have a role in migration (Geisbrecht and Montell, 2002; Cavallaro and Dejana, 2011) and *Cdh11* is specifically required for migration in some models (Kiener et al., 2006, 2009; Kashef et al., 2009; Huang et al., 2010; Kaur et al., 2012). However, our *Cdh11*-knockdown cells are able to reach the boundary of the MBO, indicating that Cdh11 is not essential for their migration. The lack of an abnormal phenotype in the MBO of the *Cdh11* mutant mouse (Manabe et al., 2000), can be attributed to early compensatory effects through other adhesive proteins (Nadeau, 2003; Barbaric et al., 2007). The phenotype can be due to other adhesion molecules being downregulated in the *Foxb1* mutant. However, that does not change, rather would reinforce, the main finding—that there are two intersecting systems, and cadherin combinations underlie one of them. For specific synapse formation, other molecules, like the nectins, are also important (Takeichi, 2007).

## Conclusions

We propose that neuronal sorting inside brain nuclei, based on cell body-to-cell body interactions and responsible for brain cytoarchitecture, is caused by adhesion-based, non-combinatorial mechanisms, one important function of which would be to keep neurons sorted according to birthdate information. Additionally, non-specific adhesion mechanisms would prevent cadherin combinations from altering the birthdate-based sorting through weaker, combination-based mechanisms. The most likely role for cadherin combinations in the developing brain is to support specific synaptogenesis through specific axonal fasciculation and final target recognition.

## Conflict of Interest Statement

The authors declare that the research was conducted in the absence of any commercial or financial relationships that could be construed as a potential conflict of interest.
